# Supplementary material for: Molecular Insights into Innate Immune Response in Captive Koala Peripheral Blood Mononuclear Cells Co-Infected with Multiple Koala Retrovirus Subtypes
Source: Pathogens. 2022 Aug 14;11(8):911. doi: 10.3390/pathogens11080911 (PMC9414840; doi:10.3390/pathogens11080911)
Supplement: Supplementary file 1 [file pathogens-11-00911-s001.zip › pathogens-1788545-supplementary.pdf]

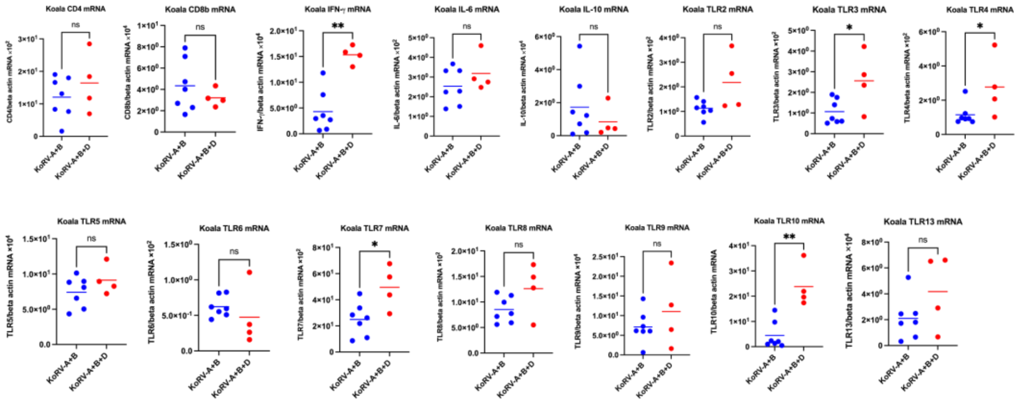


**Supplementary Figure S1.** Changes in the expression of CD4, CD8b, IFN-γ, IL-6, IL-10, TLRs (TLR2–10 and TLR13) mRNAs in koala PBMCs infected with KoRV-A+B and KoRV-A+B+D. The transcript levels were normalized against koala beta actin mRNA levels. A Mann–Whitney U test was performed to analyze the significant difference of gene expression patterns between the groups. * $p < 0.05$ and ** $p < 0.01$ indicate significant differences between the groups, and 'ns' indicates that the difference is not significant. Thick horizontal lines indicate arithmetic mean.

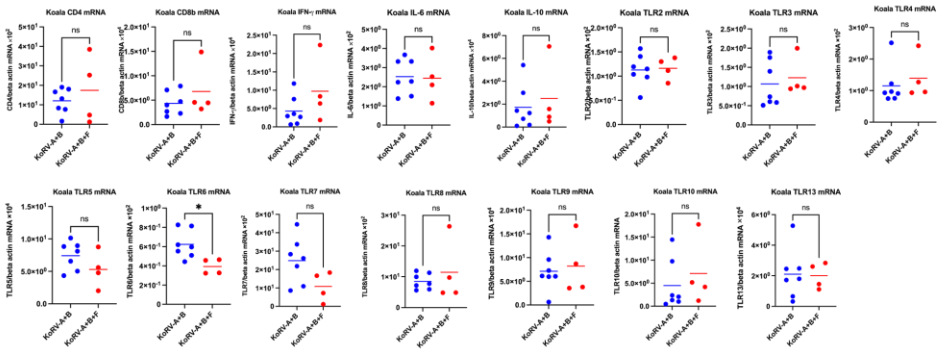


**Supplementary Figure S2.** Changes in the expression of CD4, CD8b, IFN-γ, IL-6, IL-10, TLRs (TLR2–10 and TLR13) mRNAs in koala PBMCs infected with KoRV-A+B and KoRV-A+B+F. The transcript levels were normalized against koala beta actin mRNA levels. A Mann–Whitney U test was performed to analyze the significant difference of gene expression patterns between the groups. * $p < 0.05$ indicates significant differences between the groups, and 'ns' indicates that the difference is not significant. Thick horizontal lines indicate arithmetic mean.

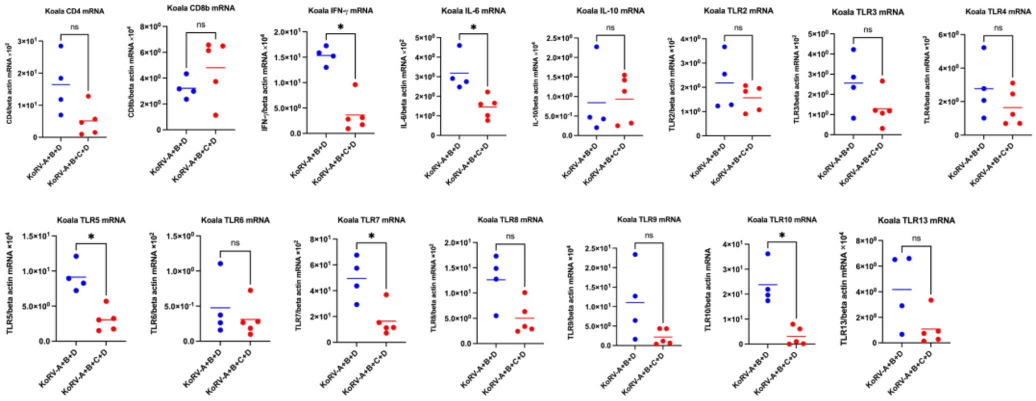


**Supplementary Figure S3.** Changes in the expression of CD4, CD8b, IFN-γ, IL-6, IL-10, TLRs (TLR2–10 and TLR13) mRNAs in koala PBMCs infected with KoRV-A+B+D and KoRV-A+B+C+D. The transcript levels were normalized against koala beta actin mRNA levels. A Mann–Whitney U test was performed to analyze the significant difference of gene expression patterns between the groups. * $p < 0.05$ indicates significant differences between the groups, and 'ns' indicates that the difference is not significant. Thick horizontal lines indicate arithmetic mean.

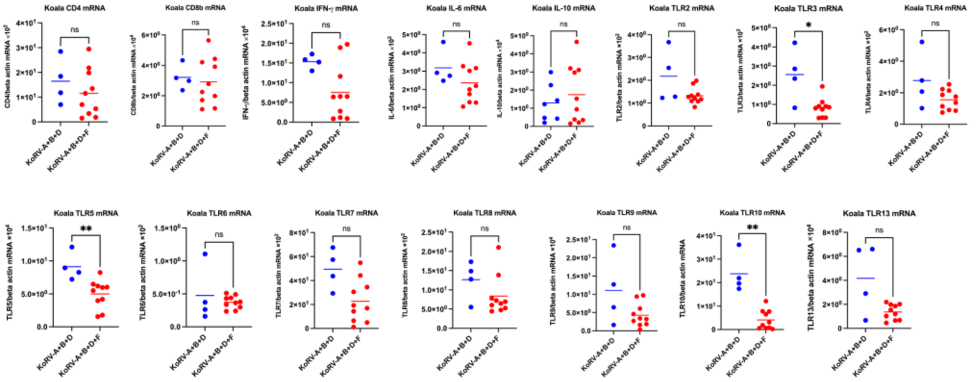


**Supplementary Figure S4.** Changes in the expression of CD4, CD8b, IFN-γ, IL-6, IL-10, TLRs (TLR2–10 and TLR13) mRNAs in koala PBMCs infected with KoRV-A+B+D and KoRV-A+B+D+F. The transcript levels were normalized against koala beta actin mRNA levels. A Mann–Whitney U test was performed to analyze the significant difference of gene expression patterns between the groups. * $p < 0.05$ and ** $p < 0.01$ indicate significant differences between the groups, and 'ns' indicates that the difference is not significant. Thick horizontal lines indicate arithmetic mean.

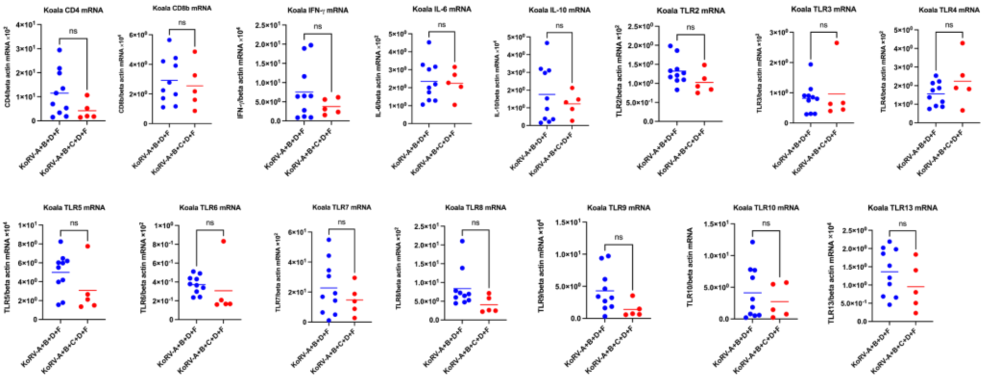


**Supplementary Figure S5.** Changes in the expression of CD4, CD8b, IFN-γ, IL-6, IL-10, TLRs (TLR2–10 and TLR13) mRNAs in koala PBMCs infected with KoRV-A+B+D+F and KoRV-A+B+C+D+F. The transcript levels were normalized against koala beta actin mRNA levels. A Mann–Whitney U test was performed to analyze the significant difference of gene expression patterns between the groups. 'ns' indicates that the difference is not significant. Thick horizontal lines indicate arithmetic mean.
